# 1892. Rise of COVID-19 Re-infections as Omicron Variant Prevailed: Implications on Monitoring the Course of the Pandemic

**DOI:** 10.1093/ofid/ofac492.1519

**Published:** 2022-12-15

**Authors:** Kassiani Mellou, Kassiani Gkolfinopoulou, Kyriaki Tryfinopoulou, Ioannis Panagoulias, Panagiota Psallida, Gerasimos Gerolymatos, Sotirios Tsiodras, Georgios Panagiotakopoulos, Dimitrios Paraskevis, Theoklis Zaoutis

**Affiliations:** Hellenic National Public Health Organization, Athens, Attiki, Greece; Hellenic National Public Health Organization, Athens, Attiki, Greece; Hellenic National Public Health Organization, Athens, Attiki, Greece; Hellenic National Public Health Organization, Athens, Attiki, Greece; Hellenic National Public Health Organization, Athens, Attiki, Greece; Hellenic National Public Health Organization, Athens, Attiki, Greece; 4th Department of Internal Medicine, University General Hospital Attikon, Medical School, National and Kapodistrian University of Athens, 12462 Athens, Greece, Athens, Attiki, Greece; Hellenic National Public Health Organization, Athens, Attiki, Greece; Hellenic National Public Health Organization, Athens, Attiki, Greece; Hellenic National Public Health Organization, Athens, Attiki, Greece

## Abstract

**Background:**

The emergence of the highly infectious Omicron variant at the end of 2021 changed the pandemic dynamics, also causing an increase in COVID-19 re-infections. Our aim was to investigate suspected re-infections and the characteristics of individuals with multiple infections since the beginning of the pandemic until early April 2022.

**Methods:**

Since the beginning of the pandemic, all COVID-19 positive tests (Rapid Antigen Tests - RAT and PCR) have been recorded in the COVID-19 National Electronic Registry. Cases were extracted and reported daily based on the first positive test for each individual (first episodes). Establishment of the Omicron variant, associated with a re-infection surge, triggered the decision for modifying case definition and applying a different methodology to include suspected re-infections in the number of daily reported cases after 3^rd^ of April. Suspected re-infections were defined as “positive PCR or RAT sample ≥90 days following a previous positive PCR or RAT”. The number of re-infections were estimated retrospectively on the Registry’s data.

**Results:**

Overall, 6,348 suspected re-infections were recorded up to 15/12/2021 (0.6% of the laboratory confirmed tests), whereas until 3/4/22 the number was increased at 115.201, raising the percentage to 4%. Distribution of the number of reinfections per ISO week is depicted in Figure 1, along with the number of first episodes. Suspected re-infections stand for 3.7% of the total number of first episodes recorded up to 3/4/22 (3,077,711), with their integration having a noticeable effect on case counting. Among them, 99.3% represent a second episode of infection and 0.7% multiple re-infections. Median age of suspected re-infections was 29 years (IQR: 19-44) (Figure 2). The median length of the time interval between two subsequent infections was 8 months (IQR: 5-12) (Figure 3).

**Figure:**
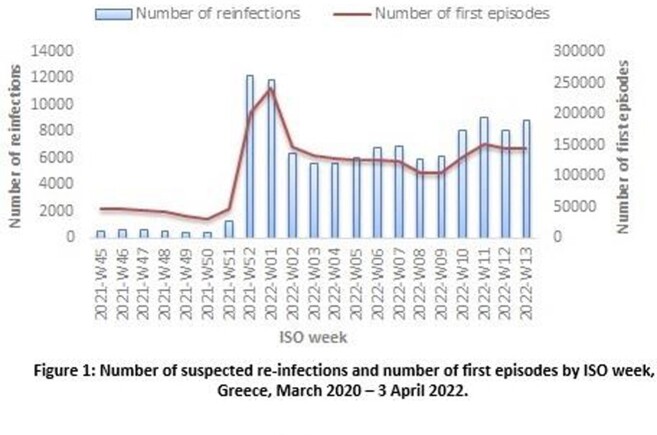

**Figure:**
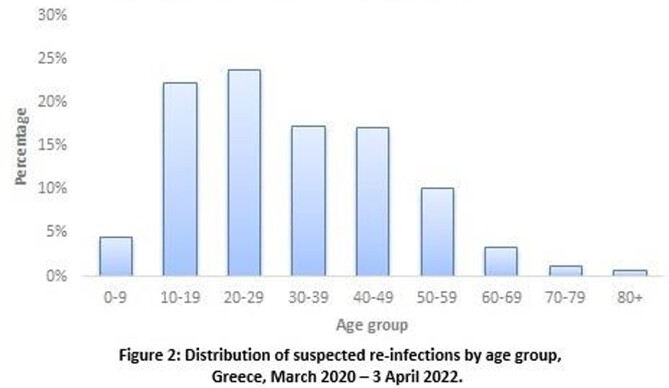

**Figure:**
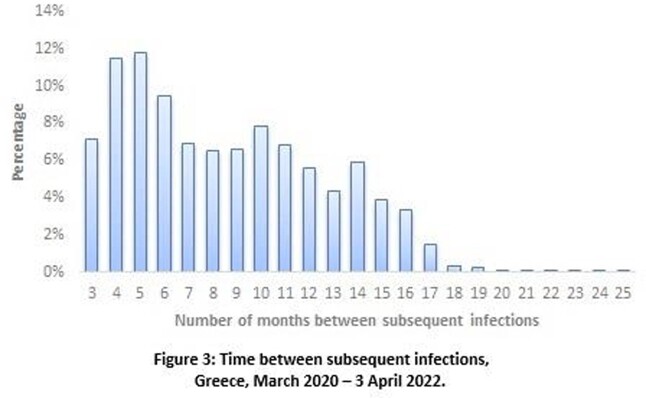

**Conclusion:**

Up until mid-December 2021, COVID-19 re-infection was uncommon, changing to higher rates after the emergence of Omicron variant, thus documenting the immune escape capacity of this variant. Besides the improvement in surveillance, knowledge about the re-infections and their characteristics provides a proxy of the immune protection from previous exposure to COVID-19, against circulating variants over time.

**Disclosures:**

**All Authors**: No reported disclosures.

